# Supplementary material for: Interactions between Bacteriophage, Bacteria, and the Mammalian Immune System
Source: Viruses. 2018 Dec 25;11(1):10. doi: 10.3390/v11010010 (PMC6356784; doi:10.3390/v11010010)
Supplement: Supplementary file 1 [file viruses-11-00010-s001.pdf]

The forgotten viruses – depicting bacteriophage interactions with human cells

Jonas D. Van Belleghem[1], Krystyna Dąbrowska[2], Mario Vaneechoutte[1] and Jeremy J. Barr[3]

[1] Laboratory Bacteriology Research, Department of Clinical Chemistry, Microbiology and Immunology, University Ghent, Medical Research Building II, Corneel Heymanslaan 10, 9000, Ghent, Belgium.

[2] Bacteriophage Laboratory, Institute of Immunology and Experimental Therapy, Polish Academy of Sciences, Weigla 12, 53-114 Wrocław, Poland

[3] School of Biological Sciences, Monash University, Melbourne, VIC, 3800, Australia

In silico **model**

The addition of anti-inflammatory phage properties in the *in silico* models, described by Leung and Weitz (2017), and Hodyra-Stefaniak *et al.* (2015), provides the phage with an increased survival rate. Therefore, we propose to expand the equation of the innate immune response (Table 1 - Equation 3), to include the effect of phages on the innate immune response (X_P). We propose to divide the initial equation describing the innate immune response (I) into two parts (Table 1 – Equation 4-9), one part describing the effect of bacteria towards the innate immune response (X_S; Table 1 – Equation 4-6) and the other one describing the effect of phages on the innate immune response (X_P; Table 1 – Equation 7-9). Furthermore, additional constraints were given to the mathematical model. When the phage concentration (P) is larger than the critical phage concentration (P_C), the decay rate (Υ_{dP}) approaches one. The critical phage concentration (P_C) is the phage concentration needed in order for the phage to induce an anti-inflammatory response and reduce the innate immune response.

Supplementary tables:

Table 1: Generalized model describing the phage-bacteria-immune response interaction. The Hodyra-Stefaniak *in-silico* model was expanded to include the anti-inflammatory properties of phages. The dynamics of bacteria, phage, innate and adaptive immunity and their interactions are described by differential equations. When the concentration of bacterial cells exceeds the threshold S_C and resources are unlimited, the concentration of innate system particles grows exponentially with a constant rate of a_I. When the phage concentration exceeds the threshold P_C and resources are unlimited, the concentration of the innate particles decreases at a constant rate of Υ_g, ultimately approaching one.

S: Growth of the bacterial population

$$\frac{dS(t)}{dt} = a_S S(t) - \rho S(t)P(t) - \kappa_{SI} S(t)I(t) - \kappa_{SB} S(t)B(t) \quad (1)$$

P: Dynamics of a free phage population

$$\frac{dP(t)}{dt} = b\rho S(t-\lambda)P(t-\lambda) - \rho S(t)P(t) - \kappa_{PI} P(t)I(t) - \kappa_{PA} P(t)A(t) \quad (2)$$

I: Innate immune response

$$\frac{dI}{dt} = \left(X_s(t) + X_P(t)\right)I(t) \quad (3)$$

X_S: Bacterial component of the innate immune response

$$X_S = \begin{cases} a_I \gamma_{gS}(t) \text{ for } S(t) \geq S_{C,} \\ -d_I \gamma_{dS}(t) \text{ otherwise} \end{cases} \quad (4)$$

Where

$$\gamma_{gS}(t) = \left(1 + \frac{S_C}{k_I}\right)\left(\frac{S(t)}{S(t) + k_I}\right) - \frac{S_c}{k_I}, \quad (5)$$

And

$$\gamma_{dS}(t) = \sqrt{1 - \frac{S(t)}{S_C}} \quad (6)$$

X_P: Phage component of the innate immune response

$$X_P = \begin{cases} -d_I \gamma_{dP}(t) \text{ for } P(t) \geq P_{C,} \\ a_I \gamma_{gP}(t) \text{ otherwise} \end{cases} \quad (7)$$

Where

$$\gamma_{gP}(t) = \left(1 + \frac{P_C}{k_I}\right)\left(\frac{P(t)}{P(t) + k_I}\right) - \frac{P_c}{k_I}, \quad (8)$$

And

$$\gamma_{dP}(t) = \sqrt{1 - \frac{P_C}{P(t)}} \quad (9)$$

A: Dynamics of adaptive immune response against phage

$$\frac{dA(t)}{d(t)} = a_A \left(\frac{P(t)}{P(t) + k_A}\right) A(t) \left(1 - \frac{A(t)}{A_{max}}\right) \quad (10)$$

B: Dynamics of adaptive immune response against bacteria

$$\frac{dB(t)}{d(t)} = a_B \left(\frac{S(t)}{S(t) + k_B}\right) B(t) \left(1 - \frac{B(t)}{B_{max}}\right) \quad (11)$$

Table 2: State variable and parameters of the models described in Table 1.

	State variable	Unit	Figure number								
			A	B	C	D	E	F	G	H	I
S	Concentration of susceptible bacteria	Cfu/ml	3000	3000	3000	3000	3000	3×10^5	3×10^5	3×10^5	3×10^5
$P(t_P)$	Concentration of phage on time point p	Pfu/ml	2×10^5	2×10^5	2×10^5	2×10^8	2×10^8	2×10^4	2×10^4	2×10^5	2×10^5
I	Concentration of particles of the innate immune response	Particles/ml	100	100	100	100	100	100	100	100	100
A	Concentration of particles of the adaptive immune response against phage	Particles/ml	-	-	-	-	-	10^4	10^4	10	10
B	Concentration of particles of the adaptive immune response against bacteria	Particles/ml	-	-	-	-	-	1000	1000	1000	1000
	**Parameters**										
a_S	Growth rate of susceptible bacteria	h^{-1}	0.7	0.7	0.7	0.7	0.7	0.7	0.7	0.7	0.7
a_I	Growth rate of innate immune response	h^{-1}	0.5	0.5	0.5	0.5	0.5	0.5	0.5	0.5	0.5
a_A	Growth rate of adaptive immune response against phage	h^{-1}	-	-	-	-	-	0.9	0.9	0.9	0.9
a_B	Growth rate of adaptive immune response against bacteria	h^{-1}	-	-	-	-	-	0.9	0.9	0.9	0.9
d_I	Decay rate of innate immune response	h^{-1}	0.5	0.5	0.5	0.5	0.5	0.5	0.5	0.5	0.5
ρ	Adsorption rate of phages by susceptible bacteria	ml cfu^{-1} h^{-1}	10^{-8}	10^{-8}	10^{-8}	10^{-8}	10^{-8}	10^{-8}	10^{-8}	10^{-8}	10^{-8}
κ_{SI}	Killing rate of bacteria versus innate immune response	h^{-1}	10^{-6}	10^{-6}	10^{-6}	10^{-6}	10^{-6}	10^{-6}	10^{-6}	10^{-6}	10^{-6}
κ_{PI}	Killing rate of phage versus innate immune response	h^{-1}	0	10^{-3}	10^{-3}	10^{-3}	10^{-3}	10^{-3}	10^{-3}	10^{-3}	10^{-3}
κ_{SB}	killing rate of bacteria versus adaptive immunity	h^{-1}	-	-	-	-	-	10^{-3}	10^{-3}	10^{-3}	10^{-3}
κ_{PA}	Killing rate of phage versus adaptive immunity	h^{-1}	-	-	-	-	-	10^{-3}	10^{-3}	10^{-3}	10^{-3}

S_C	Bacterial concentration above which innate immune response increases	Cfu/ml	100	100	100	100	100	100	100	100	100
P_C	Phage concentration above which innate immune response increases	Pfu/ml	-	10^5	-	-	10^5	-	10^5	-	10^5
Υ_g	Growth rate reduction of innate immune response		-	-	-	-	-	-	-	-	-
k_I	Bacterial concentration at which innate immune response actual growth rate is half of its maximum value	Cfu/ml	10^6	10^6	10^6	10^6	10^6	10^6	10^6	10^6	10^6
Υ_d	Decay rate reduction of innate immune response		-	-	-	-	-	-	-	-	-
b	Phage burst size	Pfu	40	40	40	40	40	40	40	40	40
λ	Latent period (average time between phage adsorption and burst	h	0.612	0.612	0.612	0.612	0.612	0.612	0.612	0.612	0.612
k_A	Phage concentration at which adaptive immune response actual growth rate is half of its maximum value	Pfu/ml	-	-	-	-	-	10^5	10^5	10^5	10^5
k_B	Bacterial concentration at which adaptive immune response actual growth rate is half of its maximum value	Cfu/ml	-	-	-	-	-	10^5	10^5	10^5	10^5
A_{max}	Maximum magnitude of A	Particles/ml	-	-	-	-	-	10^6	10^6	10^6	10^6
B_{max}	Maximum magnitude of B	Particles/ml	-	-	-	-	-	10^6	10^6	10^6	10^6
